# A Conversational, Virtual, Avatar-Led Cognitive Behavioral Therapy App Intervention for Improving the Quality of Life and Mental Health of People With Epilepsy: Protocol for a Randomized Controlled Trial

**DOI:** 10.2196/40261

**Published:** 2022-11-21

**Authors:** Frank Burbach, Francesca Lecce, Victoria M E Allen, Catherine M Porter

**Affiliations:** 1 Healios Ltd London United Kingdom

**Keywords:** epilepsy, mental health, anxiety, depression, quality of life, cognitive behavioral therapy, digital therapy, smartphone, mobile phone, app

## Abstract

**Background:**

Epilepsy is a common neurological disorder affecting about 1 in 100 people in the United Kingdom. Many individuals experience a lower quality of life as a result of their epilepsy diagnosis and are more likely to develop mental health problems, such as anxiety and depression. Medical interventions for this client group tend to focus on the treatment of seizures, whereas mental health disorders often remain undiagnosed and untreated. Early identification and treatment of mental health difficulties in people with epilepsy are vital to ensure better outcomes and improvements in quality of life.

**Objective:**

The aim of this exploratory randomized controlled trial is to evaluate whether an 8-week cognitive behavioral therapy–based intervention delivered through a mobile app—ThinkNinja for Epilepsy—is a clinically effective tool to improve quality of life, mental health, and emotional well-being in a large sample of people with epilepsy and anxiety or comorbid anxiety and depression.

**Methods:**

The study aims to recruit 184 individuals, 18 to 65 years of age, with a self-reported diagnosis of epilepsy and anxiety or comorbid anxiety and depression. Participants will be randomly assigned to the ThinkNinja for Epilepsy app condition (arm A) or the waiting-list control group (arm B). Participants in arm A will receive access to the ThinkNinja for Epilepsy app first. After 8 weeks, participants in arm B will receive the same full access to the ThinkNinja for Epilepsy app as the participants in arm A. This design will allow an initial between-subjects analysis between the two conditions as well as a within-subject analysis including all participants. The primary outcome is participants’ quality of life, measured by the 10-item patient-weighted Quality of Life in Epilepsy questionnaire. The secondary outcomes include measures of anxiety, using the 7-item Generalized Anxiety Disorder assessment; depression, using the 9-item Patient Health Questionnaire; medication adherence, using the Medication Adherence Questionnaire; and impression of change, using the Patient Global Impression of Change questionnaire.

**Results:**

Recruitment for this study began in March 2022 and was completed in October 2022. We expect data collection to be finalized by May 2023 and study results to be available within 12 months of the final data collection date. Results of the study will be written up as soon as possible thereafter, with the intention of publishing the outcomes in high-quality peer-reviewed journals.

**Conclusions:**

This study aims to determine the clinical efficacy and safety of the ThinkNinja for Epilepsy intervention at improving the quality of life, mental health, and emotional well-being of people with epilepsy. The findings from our study will hopefully contribute to addressing the critical gap in universal provision and accessibility of mental health and emotional well-being support for people with epilepsy.

**Trial Registration:**

ISRCTN Registry 16270209 (04/03/2022); https://www.isrctn.com/ISRCTN16270209

**International Registered Report Identifier (IRRID):**

DERR1-10.2196/40261

## Introduction

### Background

Approximately 1 in 100 people in the United Kingdom have a diagnosis of epilepsy, with around 87 people being diagnosed every single day. This level of prevalence equates to approximately 500,000 people in the United Kingdom having an official diagnosis of epileptic seizures [1]. Many individuals are subsequently diagnosed with anxiety after their epilepsy diagnosis and also experience lower quality of life [2-4]. The fear of having a seizure is reported to be one of the most common causes for anxiety in those with epilepsy [5].

Research has found that epilepsy is comorbid with anxiety and depression, and that people with epilepsy are more likely to experience these mental health issues than the general population [6]. It is estimated that the lifetime prevalence of depression in people with epilepsy is around 55%. Comorbid anxiety and depression affect 20.2% and 22.9 % of people with epilepsy, respectively [7]. Depression and anxiety co-occur in 16% to 19.9% of cases, depending on studies [8,9]. People with epilepsy are also at increased risk for suicidal ideation and behaviors [10,11]. It has been hypothesized that psychiatric comorbidities in people with epilepsy might be the consequence of a potential common underlying biological etiology [11], a possible side effect of antiepileptic drugs, and the psychological impact and hopelessness resulting from living with a chronic health condition [12,13].

Astonishingly, there is still little research into this issue, its possible causes, or possible treatment solutions. Medical interventions for this client group tend to focus on the treatment of seizures, though it is equally important that doctors can recognize and address symptoms of anxiety and depression. Depression can increase the frequency of epileptic seizures by means of sleep deprivation. Panic disorder and phobic disorders, such as agoraphobia, are common in epilepsy patients. These are often the result of poor seizure control or the fear of having a seizure [14]. People with epilepsy and anxiety tend to have more severe seizures and a lower quality of life [15]. Effective epilepsy management should incorporate the early detection of psychological disorders and the promotion of appropriate interventions [2] in order to improve the quality of life of people with epilepsy [16,17].

Smartphone apps for mental health have seen considerable growth in recent years [18]. However, few apps have been specifically developed for people with epilepsy, much less an app focusing on the mental health, emotional well-being, and quality of life of people with epilepsy. The majority of the smartphone apps developed for epilepsy are for seizure management and seizure diaries [19].

Cognitive behavioral therapy (CBT) is one of the most thoroughly investigated and effective alternatives to medication for mental health issues, such as anxiety and depression [20,21], and it is recommended in England’s National Institute for Health and Care Excellence (NICE) clinical guidelines [22-24]. Although internet-based CBT programs have existed for some time, their sometimes-inadequate design and usability have inhibited their popularity and advancement [25,26].

In a recent systematic review (F Lecce, CR Smith, and FR Burbach, unpublished, 2022), we identified six digital mental health interventions for people with epilepsy, but only one of them—Emyna—was a fully automated epilepsy-specific CBT-based program. Emyna is a 180-day internet intervention that requires no clinician support and is accessed via a secure, password‐protected website from a computer or smartphone. In a recent clinical trial in Germany, Meyer and colleagues [27] examined the effectiveness of this fully automated internet-delivered treatment in reducing symptoms of depression and anxiety and improving quality of life in a sample of 200 people with epilepsy and comorbid depression. The authors found that participants experienced significantly greater improvement in depression, anxiety, and quality of life compared to the control group and concluded that the intervention was effective when used adjunctively to usual care.

### Aims of This Study

In this exploratory randomized controlled trial (RCT), we will evaluate whether an 8-week CBT-based intervention, delivered through ThinkNinja for Epilepsy (Healios Ltd), is a clinically effective tool for improving the quality of life, mental health, and emotional well-being in a large sample of people with epilepsy.

We hypothesize the following:

1. There will be an improvement in participants’ self-reported quality of life scores, as measured by the 10-item patient-weighted Quality of Life in Epilepsy questionnaire (QOLIE-10-P) [28], as a result of the ThinkNinja for Epilepsy intervention.
2. There will be an improvement in participants’ self-reported anxiety, depression, impression of change, and medication adherence, as measured by the 7-item Generalized Anxiety Disorder assessment (GAD-7) [29], the 9-item Patient Health Questionnaire (PHQ-9) [30], the Patient Global Impression of Change questionnaire (PGIC) [31], and the Medication Adherence Questionnaire (MAQ) [32], respectively, as a result of the ThinkNinja for Epilepsy intervention.
3. There will be a positive association between the level of engagement with the app and the primary and secondary outcomes.

## Methods

### Ethics Approval

The study was approved on August 20, 2021, by the Cambridge East Research Ethics Committee (REC reference No. 21/EE/0128). Initial recruitment will occur via social media, and interested participants will complete a brief online screening questionnaire and provide their informed consent before being randomized and given access to the app via our secure clinical platform. Data will be directly collected via the app, and clinical scales will also be completed via our secure platform. Healios is registered with the National Health Service (NHS) Data Security and Protection Toolkit standards for patient data and achieves the required level-2 information governance standards for provision of clinical services on behalf of the NHS. Healios complies with the UK General Data Protection Regulation requirements regarding the collection, storing, and processing of clinical and study data. At the end of the study, all data will be pseudonymized prior to statistical analyses. Participants will not be compensated for their participation in the study. This study was registered at the ISRCTN Registry (16270209) on March 4, 2022.

### Study Setting

The study intends to recruit 184 participants. Individuals interested in taking part in the research have to be residents in the United Kingdom, aged between 18 and 65 years, and have a self-reported diagnosis of epilepsy. Recruitment of participants will be conducted via advertisements on epilepsy charity websites and mailing lists as well as social media platforms, such as Facebook and Twitter. Participants will be able to download ThinkNinja for Epilepsy on their smartphones and use it independently, with additional support provided by the research team where needed. It is expected that participants will use the ThinkNinja for Epilepsy app in their own private time.

### Study Design

Participants will be randomly assigned to a waiting-list control group or will receive full access to the ThinkNinja for Epilepsy smartphone app. A mixed study design will allow for an initial between-subjects analysis between the two condition arms—those who receive the app straight away versus those who wait for access—as well as a within-subject analysis of those who complete the intervention; this includes those who receive the intervention straight away as well as those who receive it after the waiting period. The study adheres to relevant ethical guidelines from the British Psychological Society [33]. In addition, the completeness, content, and quality of the study protocol is reported in line with the Standard Protocol Items: Recommendations for Interventional Trials guidelines [34].

### Eligibility Criteria

All individuals who meet the inclusion criteria will be eligible to participate. Inclusion and exclusion criteria are outlined in Textbox 1. Participants will have a self-reported diagnosis of epilepsy received at least 6 months before applying, and they will be encouraged to share photographs of their current medication, letter, or report from their health care provider. There will be no restrictions for participants to access any other form of psychological services during the study; however, they will be asked to disclose this information while completing the measures throughout the study. Participants will require access to a personal Apple iOS or Android smartphone and will be able to download the ThinkNinja for Epilepsy app from the iTunes store or Google Play. The app is completely free for participants to download using Wi-Fi, and there are no in-app purchases. However, downloading the app via data roaming may incur additional charges as per the user’s contract fees; therefore, the use of Wi-Fi is encouraged.

**Study inclusion and exclusion criteria.**

**Inclusion criteria**

- Adults aged 18-65 years
- UK resident
- Fluent in English
- Scoring ≥5 on the 7-item Generalized Anxiety Disorder assessment (GAD-7; mild anxiety) at screen-in
- Willing and able to receive notifications and SMS text and email messages
- A confirmed epilepsy diagnosis (6 months minimum time since diagnosis; suspected cases are not permitted); diagnosis to be confirmed, ideally by participants submitting photographs of their current medication, letter, or report from their health care provider
- Stable epilepsy medication and anxiety or depression medication regimens (antiepileptic, antidepressant, anxiolytic drug, etc); stable medication regimen for this study refers to no change in medication in the last 4 weeks; questions to cover this at all data collection time points; should participants change medication during the study period, this will not affect their inclusion, however, this will be explored in the analysis

**Exclusion criteria**

- Scoring <5 on the GAD-7 at screen-in
- Having a score of ≥20 on the 9-item Patient Health Questionnaire, indicating severe depression at screening, or if they answer, “more than half of the days” or “nearly every day” to the question “Over the last 2 weeks, how often have you been bothered by thoughts that you would be better off dead or of hurting yourself in some way?”
- Sensitivity to mobile phone screen exposure
- Currently receiving counseling or psychological therapy; however, they will not be excluded if they seek support during the study; questions in measures to reflect this
- Individuals involved in current or ongoing research
- Pregnant or gave birth in the past 12 months
- Diagnosis of a severe mental illness (eg, severe depression including suicidal ideation, schizophrenia, bipolar disorder, psychosis, personality disorder, posttraumatic stress disorder, and substance misuse)
- Severe learning disability and individuals requiring a carer for their epilepsy
- Does not have access to a smartphone (ie, iPhone with iOS 13 or greater capabilities or an Android phone with OS 7 or greater capabilities)

### Recruitment

Potential participants will be able to click a link in the advertisement, which will lead them to a short contact form collecting their name, date of birth, home address, and email address. Personal information will be stored in our secure system. The GAD-7 will be used to screen potential participants’ suitability for inclusion, with scores of 5 or greater as the cut point for mild anxiety. The PHQ-9 will also be used as a screening questionnaire. If we identify severe depression (score ≥20) or significant suicidal risk, potential participants will be excluded from the study and advised to seek professional help via the NHS. Significant suicidal risk is identified if they answer, “more than half of the days” or “nearly every day” to the PHQ-9 question “Over the last 2 weeks, how often have you been bothered by thoughts that you would be better off dead or of hurting yourself in some way?” We will also include contact details for national crisis support helplines in the return email to ensure that participants are aware of the support they can receive from national helplines. The GAD-7 and the PHQ-9 are well-researched measures with sound psychometric properties [35]. The same measures will also be used throughout the study at the specified time points (T) to assess any change in participants’ self-reported anxiety and depression scores.

To mitigate risk to implementation, we aim to complete baseline measures and enrollment with all participants over a 2- to 4-week period. Opt-in consent forms will first be distributed to all participants.

### Participant Classification

Data will be input into the R Minirand program (version 0.1.3; R Foundation for Statistical Computing) for randomization, and participants will be assigned to either the ThinkNinja for Epilepsy app arm (condition A) or the wait-list control arm (condition B) using minimization techniques. The prognostic factors over which the data will be minimized include age, sex at birth, and education level. Each prognostic factor will be minimized over two distinct categories: age (over 40 and under 40), sex at birth (male and female), and education level (below degree level and degree plus). Prognostic factors will have equal weighting in randomization. As there is not a requirement to blind this trial (ie, both clinicians and patients will be aware of whether they receive treatment or not), a deterministic approach is satisfactory; therefore, the significance level on which participants will be randomized is *P*>.99.

### Intervention

Healios has developed a CBT-based app called ThinkNinja for Epilepsy. ThinkNinja for Epilepsy is designed to support users’ individual situations and to address mental health challenges with weekly mini modules. These are guided by an automated virtual assistant, the Wise Ninja, combined with interactive screens that are designed to cover an 8-week period, delivered at the user’s pace. Each week of the program includes three sessions, and users have to complete each session before being able to progress to the next one. Every week, new content is released and three more sessions become available to the user, allowing them time to digest information, develop their understanding, and practice coping and CBT skills to manage their epilepsy and mental health. Moreover, the structured epilepsy-specific 8-week program provides tools for monitoring epileptic seizures as well as ways of helping individuals understand and improve their mental health and emotional well-being (Table 1).

The first week includes an overview of the program and psychoeducation regarding epilepsy and mental health. Participants are then introduced to the “seizure diary” and are helped to think about triggers and warning signs for seizures. Week 2 introduces the main principles of CBT, and participants are encouraged to watch two short videos about anxiety and low mood. Participants learn about “automatic thoughts” and how these can impact on their mood. They learn to assess their own thoughts and how these might not always be accurate or helpful. Week 3 explores maladaptive coping strategies (ie, safety behaviors) and how to spot them. In week 4, participants further develop their CBT skills and learn about the importance of facing their fears, set daily quests, and practice using positive statements. Users are also introduced to some relaxation skills, including breathing exercises and “grounding” techniques. In weeks 5 and 6, participants learn ways of challenging difficult thoughts. They are encouraged to evaluate their beliefs and cognitive biases and begin to develop alternative thoughts via the “thought diary.” They are also introduced to further relaxation and mindfulness exercises. Week 7 focuses on practicing alternative thoughts and stress reduction techniques. Week 8 involves reflecting on the progress made and relapse prevention as well as thinking about how to share this with their support network.

**Table 1:** Overview of the 8-week program.

Week	Session 1	Session 2	Session 3
1	Introduction and resources	My epilepsy diary	Setting goals
2	Introduction to CBT^a^	Anxiety and low mood videos	Thinking traps
3	Safety behaviors	The spotlight	Unhelpful coping
4	Formulation	Hot cross bun	Grounding techniques
5	Challenging thoughts	Thought challenger	Relaxation
6	Snapshots (gratitude journal)	Thought diary introduction	Thought diary 1
7	Thought diary 2	Breaking it down	Confidence boost
8	Moving on	Keeping calm	Staying well

^a^CBT: cognitive behavioral therapy.

As part of augmenting the 8-week structured self-management program, within the app, there are two “step-up” levels to allow the user to access further clinical support via interaction with a trained clinician. Details of the step-up levels are as follows:

1. Step-up level 1 is a continuation of the CBT intervention and is a text-based feature enabling a trained mental health coach to perform live problem-solving, perform assessment of need, and signpost additional support where required, all via a text-chat interface within the app. Participants may request a text-based chat with a clinician through a designated button on the home screen. Text support will be available to participants Monday to Friday, 9 AM to 6 PM, with all requests responded to within a maximum of 24 hours. For the 8-week duration of the active participation section of the study, participants will have access to the step-up level-1 feature, if necessary.
2. Step-up level 2 is a video-based, brief, goal-focused continuation of the CBT intervention involving up to three live video-based sessions with a clinician to learn skills to manage symptoms of anxiety and low mood using CBT techniques. The level-2 step-up is only possible via accessing level 1 and is determined by the clinician based on a needs assessment. Step-up level 2 will be available to participants for the 8-week duration of the active participation section of the study.

ThinkNinja for Epilepsy was created with a range of external inputs. Healios CBT therapists and clinical psychologists developed the CBT clinical architecture of the app, in order to ensure clinical accuracy in accordance with the NICE clinical guidelines, as well as appropriate tone and language. The CBT architecture was then approved by independent UK psychologist experts as suitable for supporting both mental health and emotional well-being, as well as supporting symptoms of anxiety and low mood. Epilepsy field experts, such as neurologists, neuropsychiatrists, and clinical psychologists, were then consulted during the creation of the epilepsy-specific content in order to ensure that the app could be as valuable as possible to persons with epilepsy. As part of the co-design with users, four user focus groups—18 participants across three face-to-face groups and one online group—were conducted to provide input into app design concepts covering look, feel, and tone suitability. During the app technical build process, an online group of 6 users was set up to provide weekly input into each weekly development “sprint” of new app features, covering the tone and language of the content as well as the interactivity and usability of the app. Real-time feedback incorporated into the app on a weekly basis ensured that the development phase of the project had continued input from independent individuals.

### Outcomes and Variables

**Figure 1:**
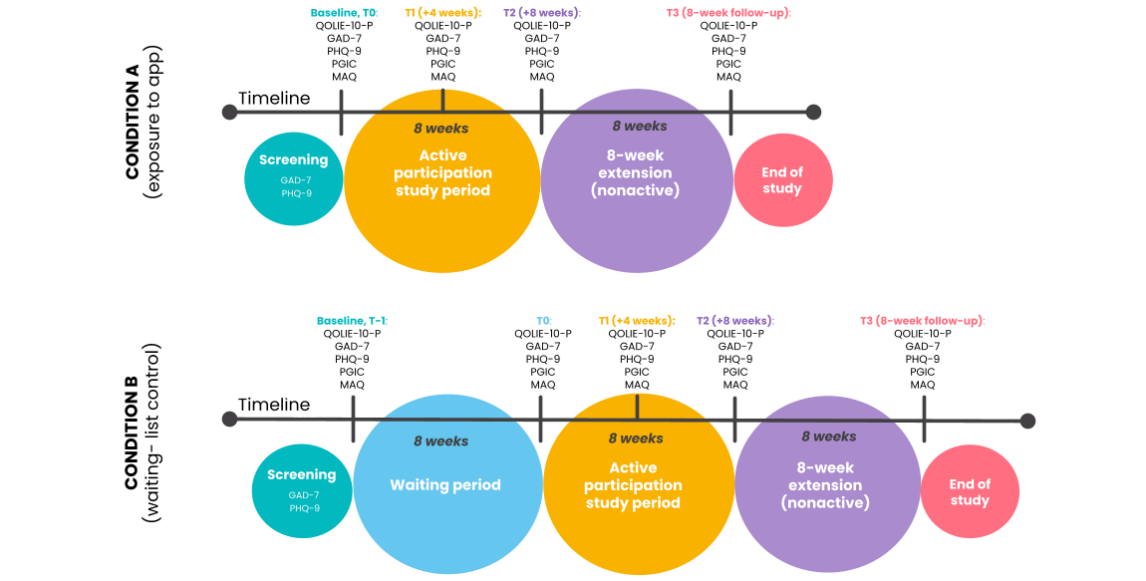
Condition A and B timelines. GAD-7: 7-item Generalized Anxiety Disorder assessment; MAQ: Medication Adherence Questionnaire; PGIC: Patient Global Impression of Change questionnaire; PHQ-9: 9-item Patient Health Questionnaire; QOLIE-10-P: 10-item patient-weighted Quality of Life in Epilepsy questionnaire; T: time point.

Figure 1 shows details of the measures and the time points at which they will be collected. The primary outcome is the QOLIE-10-P score.

Data will be collected at baseline (T0, plus T–1 for wait-list participants), T1 (4 weeks after commencement of the ThinkNinja for Epilepsy intervention), T2 (8 weeks after commencement of the intervention), and T3 (8 weeks following completion of the active period of the study) via participant-completed questionnaires.

The secondary outcomes include anxiety (GAD-7), depression (PHQ-9), patients’ global impression of change (PGIC), and self-reported medication adherence (MAQ). All measures to be collected at T–1, T0, T1, T2, T3, and weekly were chosen due to their suitability, reliability, validity, and established sensitivity to change for people with epilepsy and for evaluating changes in quality of life, anxiety, and depression levels. The measures chosen are also balanced with consideration to minimizing burden on both participants and researchers and maximizing data quality. All T–1, T0, T1, T2, T3, and weekly measures will be completed online and will be self-reported by the participants. In addition, demographic characteristics, including age, gender, and ethnicity, will be self-reported by participants at baseline [36].

At each time point, bespoke questions will also be included to cover any change in medications and any psychological support services the participant may have accessed during the study, such as services from their local NHS, including mental health services covering counseling or therapy. These will be self-reported by the participant. To minimize potential self-reporting inaccuracies, the participant will be asked to recall any additional support over the previous 3 months at T–1, T0, T1, T2, and T3, instead of recalling any additional support over the full study period at completion.

Other measures captured within the app at various frequencies via user input and app tracking mechanisms are as follows:

1. Usage data to determine fidelity of the intervention and how closely the participants complied with the recommended guidance, covering which elements of the app they use the most and for how long they use different elements of the app.
2. Goal-based achievement for those participants who set goals.
3. Mood and anxiety scores and activity ratings throughout the study.
4. Skill practice implementation data.

### Study Procedure

Participants randomly assigned to condition A will receive access to the ThinkNinja for Epilepsy app first. Participants will receive guidance on the expected frequency and duration of app use and will be encouraged to use the app two to three times per week for the duration of the 8-week study period. After the active 8-week period of the study is completed, participants may use the app on their own accord, as much as they like, for the remaining 8-week duration of the study.

**Figure 2:**
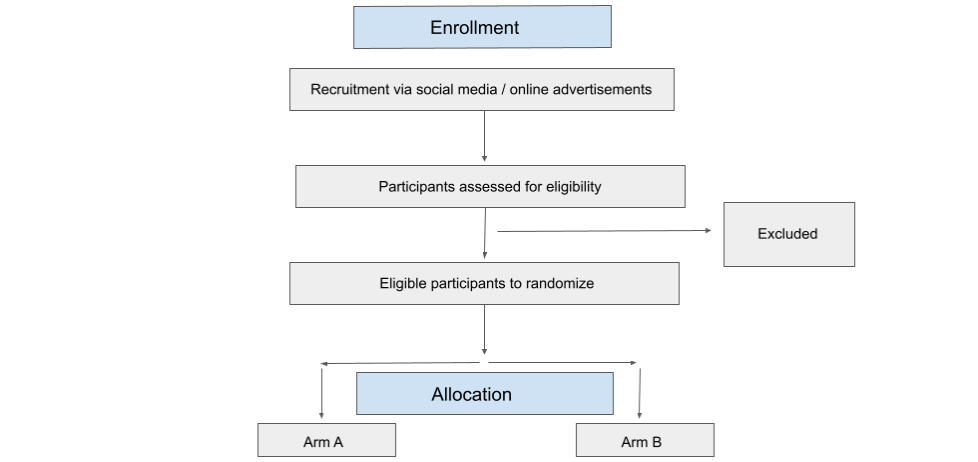
Participant flow. Arm A participants will receive access to ThinkNinja for Epilepsy app first. Arm B participants will be the waiting-list control group.

Step-ups will only be available for the 8-week active participation period of the study. Participants will be reminded via push notifications to use the app if they have not used it in the previous 3 to 7 days. Although participants will be able to disable app notifications as is standard through their device’s operating systems settings menu, we will request that they remain enabled for the duration of the study in order to facilitate communication with the research team. Participants assigned to condition B will receive the same full access to the ThinkNinja for Epilepsy app as the participants in condition A after 8 weeks. The study procedures and timing are summarized in Figures 1 and 2.

We considered performing weekly tracking of anxiety and mood for the control arm participants but, on balance, decided that for this exploratory study, the potential adverse effects on engagement due to perceived burden outweighed the advantages of collection of weekly data. The weekly measures will be collected as part of the CBT-based intervention, as is common in the delivery of CBT; this will allow us to monitor clinical change and potential risk as part of the intervention. Participants will be able to report any app technical issues via a Healios study email address and telephone number. The research team will make every effort to locate participants lost to follow-up using text and email.

### Sample Size

A formal sample size calculation was based on assumptions of QOLIE-10-P means and SDs from earlier studies [37,38]. It was performed using a 2-tailed *t* test of equal means at a significance level of .05 and a power of 80% for an estimated medium effect size (Cohen *d*=0.5). All calculations were done using the software nQuery (version 7.0; Statsols) [39]. We intend to recruit 184 people—92 participants in each arm—so that we can accommodate a dropout rate of 30%.

The chi-square method was used to determine the power for structural equation modeling (SEM) [40] using the associated online power calculator [41]. It was determined that with a sample size of 184 (see above) and a *P* value of .05, the proposed 11-parameter SEM analysis would be powered at 0.705 (70.5%).

### Data Analysis

The analysis will be performed in two phases. The first phase of analysis will occur after completion of the 8-week (T2) outcome measures, when all participants in arm A have completed the 8-week program and can be compared to the waiting-list control participants (arm B). The second phase of analysis with the pooled data will occur after the completion of the 8-week follow-up period (T3) outcome measures for all participants.

The following patient cohorts will be used in the analysis:

1. The per-protocol study cohort will include all allocated participants with at least 80% completed questionnaire data, including a completed QOLIE-10-P score, at baseline (T0 ThinkNinja for Epilepsy group and T–1 wait-list group) and at 8 weeks (T2 ThinkNinja for Epilepsy group and T0 wait-list group).
2. The intention-to-treat study cohort will include all allocated participants.

Available information on screened participants who were not randomized will be summarized.

Descriptive statistics will be produced for the distribution of all variables, including baseline demographic variables, app use, and engagement variables, as well as for each outcome variable at each time point. The change in each outcome from baseline will be reported in terms of mean and SD. An analysis of reliable and clinical change will be produced for the GAD-7 and PHQ-9 scores in order to determine the percentage of those who had clinical and reliable change (ie, clinical recovery) after taking part in the intervention.

The primary outcome analysis (ie, quality of life) will be performed for the overall cohort, both unadjusted and adjusted for baseline characteristics, app use, and app content variables. We will be comparing changes in quality of life (ie, QOLIE-10-P scores) from baseline to completion of the 8-week program.

The secondary outcome analysis will constitute analyzing the change in GAD-7 (ie, anxiety) and PHQ-9 (ie, depression) scores. In addition, we will be reporting on the results of the MAQ and PGIC, using descriptive statistics.

In addition to this, a sensitivity analysis that uses both the primary and secondary outcomes will be performed. To explore both between-subjects (ie, arm A vs arm B) and within-subject (ie, T0 vs T1 vs T2 vs T3) differences, a series of repeated-measures (2 × 4) analyses of variance (ANOVAs) will be performed in the first instance with both the primary outcome measure (ie, QOLIE-10-P score) and the secondary outcome measures (ie, GAD-7, PHQ-9, PGIC, and MAQ scores) as dependent variables. All of these ANOVA tests will be powered at 0.80 (*P*=.05). The output from this series of ANOVAs will permit the necessary sensitivity analyses to be completed.

Further exploratory subgroup analyses will be performed for the following:

1. Change in primary and secondary outcomes for subgroups defined by baseline characteristics and demographics, such as age and gender.
2. Change in primary and secondary outcomes stratified by app use, videoconference use, and text support use.
3. Engagement variables: low versus high frequency and duration of app use.

Responder and nonresponder groups will be identified on the basis of changes in primary and secondary outcomes, and these groups will be characterized in terms of baseline characteristics, demographics, and app use variables. Descriptive statistics will be produced, and a regression model will be performed to identify variables associated with responder status. An SEM model will then be developed where baseline characteristics will be independent variables, app use and content variables will be mediating latent variables, and quality of life will be the dependent variable. Regression coefficients will be reported with 95% CIs. These analyses will be performed as an intention-to-treat analysis, using all randomized participants with imputation of missing end-of-study (T2) observations, with the exception of the sensitivity analyses, which will be per-protocol analyses.

## Results

Recruitment for this study started in March 2022 and was completed in October 2022. Considering that participants who were randomized to arm B will take 16 weeks to complete the study (Figure 1), we expect data collection to be finalized by May 2023 and study results to be available within 12 months of the final data collection date. Results of the study will be written up as soon as possible thereafter, with the intention of publishing the outcomes in high-quality peer-reviewed journals.

## Discussion

### Overview

This exploratory RCT aims to investigate whether an 8-week CBT-based intervention—ThinkNinja for Epilepsy—delivered through a smartphone app is clinically effective in improving the quality of life, mental health, and emotional well-being of people with epilepsy and concurrent mental health difficulties. We hypothesize that there will be an improvement in participants’ self-reported quality of life, anxiety, depression, impression of change, and medication adherence as a result of the 8-week CBT-based intervention, with the outcomes being positively associated with participants’ levels of engagement with the app.

Despite the link between epilepsy and mental health difficulties being well known, current medical interventions tend to focus on seizure management. As a result, mental health difficulties in the context of epilepsy are too often overlooked and untreated, with detrimental outcomes on people’s quality of life.

CBT is a well-researched psychological intervention that has proved to be effective in reducing symptoms of anxiety and depression in people with epilepsy, and it is recommended in the NICE guidelines [42].

More recently, digital mental health interventions, including CBT-based programs, have become quite popular and have proved to be effective in managing and preventing mental health disorders in people suffering from chronic health conditions. Sasseville et al [43] recently conducted a rapid literature review aimed at determining the effectiveness of digital mental health interventions for people with a concomitant chronic disease. The authors concluded that digital mental health interventions are effective and safe for people with chronic health conditions, but further studies are required in order to provide precise recommendations to specific clinical populations.

To the best of our knowledge, only a few digital interventions and smartphone apps are currently available for people with epilepsy; these typically offer tools to manage seizures, such as seizure diaries [19], with only a few focusing on mental health [27].

ThinkNinja for Epilepsy appears to be the first CBT-based digital mental health smartphone app that is specifically designed to support people with epilepsy. The findings from our study will hopefully contribute to addressing the critical gap in the universal provision and accessibility of mental health and emotional well-being support for people with epilepsy.

### Limitations

We note the following limitations to the proposed study. First, participants will know that they will be able to start the intervention after an 8-week waiting period if they are randomized to the waiting-list control arm. We considered that this would potentially result in either improved mood and motivation or the opposite. This cannot be controlled for, but we will capture further baseline data (T0) when the waiting-list participants move to the active arm and start the intervention. We will also explore this in our qualitative evaluation upon completion of the study. Second, this study is based in the United Kingdom; therefore, the results of this study may have reduced generalizability to other cultures where mental health support is limited due to issues such as social stigma. Moreover, recruitment of participants will take place via advertisements on social media platforms, such as Facebook and Twitter, as well as on epilepsy charities’ websites. This may result in selection bias toward motivated participants who are likely to be better educated about their condition and able to afford mobile technologies. These types of participants may be more likely to benefit from technology-based interventions compared to patients who do not engage with social media. Therefore, care should be taken when generalizing the results of this study to the wider epilepsy population. Our study will only include participants with a self-reported diagnosis of epilepsy, as they are volunteers recruited through charities and social media platforms. We acknowledge this as a limitation and, if this study is successful, we would envisage our next trial to be held within primary care and specialist neurology services, with all referrals being made by NHS clinicians. Similarly, self-reported questionnaires will be used to assess outcomes.

We also acknowledge that participants’ ability to access other forms of psychological services during the study is a potential limitation. However, they will be asked to disclose this information while completing the measures throughout the study and it will be accounted for in our analyses. Participants may not wish to disclose this information, but this is an unavoidable limitation of this study.

### Conclusions

This study is a preliminary investigation into an underdeveloped area with significant potential for future scaling of psychological interventions for people with epilepsy. This study will attempt a sophisticated analysis of factors related to engagement and outcomes, which will inform future research and app intervention development. We envisage that this study will lead to further investigation of the ThinkNinja for Epilepsy app within clinical services.

## Abbreviations

ANOVA: analysis of variance
CBT: cognitive behavioral therapy
GAD-7: 7-item Generalized Anxiety Disorder assessment
MAQ: Medication Adherence Questionnaire
NHS: National Health Service
NICE: National Institute for Health and Care Excellence
PGIC: Patient Global Impression of Change questionnaire
PHQ-9: 9-item Patient Health Questionnaire
QOLIE-10-P: 10-item patient-weighted Quality of Life in Epilepsy questionnaire
RCT: randomized controlled trial
REC: Research Ethics Committee
SEM: structural equation modeling
T: time point (in the context of T–1, T0, T1, T2, and T3)

## Data Availability

The study described in this protocol will gather confidential and sensitive data. However, to support scientific transparency, data can be made available upon request to the corresponding author.
